# (4-Methyl­phen­yl)[1-(4-methyl­phen­yl)-3-(5-nitro-2-fur­yl)-1*H*-pyrazol-4-yl]methanone

**DOI:** 10.1107/S1600536809047758

**Published:** 2009-11-14

**Authors:** Jia Hao Goh, Hoong-Kun Fun, Nithinchandra, N. Satheesh Rai, B. Kalluraya

**Affiliations:** a X-ray Crystallography Unit, School of Physics, Universiti Sains Malaysia, 11800 USM, Penang, Malaysia; b Department of Studies in Chemistry, Mangalore University, Mangalagangotri, Mangalore 574 199, India

## Abstract

In the title pyrazole compound, C_22_H_17_N_3_O_4_, an intra­molecular C—H⋯O contact generates a seven-membered ring, producing an *S*(7) ring motif. The furan and pyrazole rings are essentially planar [maximum deviations = 0.004 (1) and 0.004 (2) Å, respectively] and are almost coplanar, making a dihedral angle of 3.75 (10)°. One of the methyl­phenyl groups is inclined to the pyrazole ring, as indicated by the dihedral angle of 48.41 (9)°. In the crystal structure, mol­ecules are linked into chains along [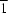
10] by C—H⋯O contacts. The crystal structure is further stabilized by π–π inter­actions [centroid–centroid distance = 3.4437 (10) Å].

## Related literature

For general background to and applications of the title compound, see: Kalluraya *et al.* (1994 ▶); Rai & Kalluraya (2006 ▶); Rai *et al.* (2008 ▶); Sridhar & Perumal (2003 ▶). For hydrogen-bond motifs, see: Bernstein *et al.* (1995 ▶). For a closely related structure, see: Goh *et al.* (2009 ▶). For the stability of the temperature controller used for the data collection, see: Cosier & Glazer (1986 ▶).
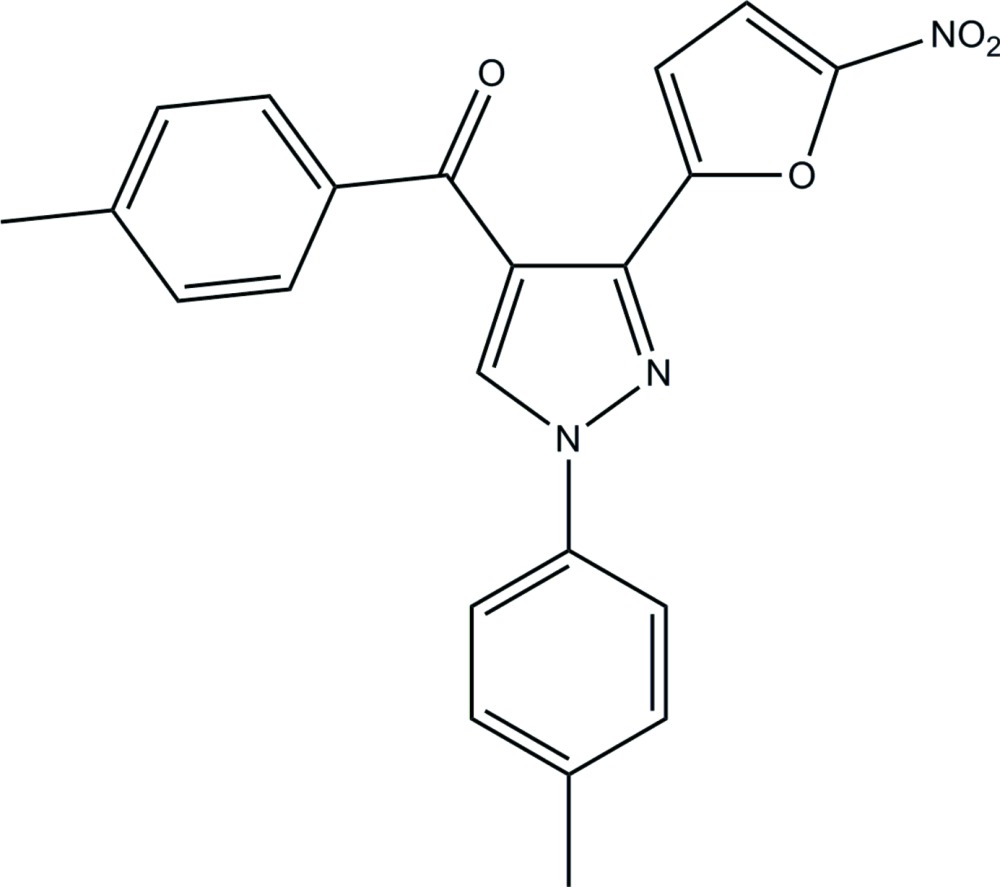


## Experimental

### 

#### Crystal data

C_22_H_17_N_3_O_4_
*M* *_r_* = 387.39
Triclinic, 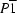

*a* = 9.6398 (2) Å
*b* = 9.9160 (2) Å
*c* = 10.1815 (2) Å
α = 88.051 (1)°
β = 85.930 (1)°
γ = 70.495 (1)°
*V* = 915.01 (3) Å^3^
*Z* = 2
Mo *K*α radiation
μ = 0.10 mm^−1^
*T* = 100 K
0.39 × 0.23 × 0.11 mm

#### Data collection

Bruker SMART APEXII CCD area-detector diffractometer
Absorption correction: multi-scan (**SADABS**; Bruker, 2005 ▶) *T*_min_ = 0.963, *T*_max_ = 0.989
21316 measured reflections
5261 independent reflections
4131 reflections with *I* > 2σ(*I*)
*R*_int_ = 0.032

#### Refinement

*R*[*F* ^2^ > 2σ(*F* ^2^)] = 0.060
*wR*(*F* ^2^) = 0.144
*S* = 1.08
5261 reflections
264 parameters
H-atom parameters constrained
Δρ_max_ = 0.51 e Å^−3^
Δρ_min_ = −0.29 e Å^−3^

### 

Data collection: *APEX2* (Bruker, 2005 ▶); cell refinement: *SAINT* (Bruker, 2005 ▶); data reduction: *SAINT*; program(s) used to solve structure: *SHELXTL* (Sheldrick, 2008 ▶); program(s) used to refine structure: *SHELXTL*; molecular graphics: *SHELXTL*; software used to prepare material for publication: *SHELXTL* nd *PLATON* (Spek, 2009 ▶).

## Supplementary Material

Crystal structure: contains datablocks global, I. DOI: 10.1107/S1600536809047758/tk2571sup1.cif

Structure factors: contains datablocks I. DOI: 10.1107/S1600536809047758/tk2571Isup2.hkl

Additional supplementary materials:  crystallographic information; 3D view; checkCIF report

## Figures and Tables

**Table 1:** Hydrogen-bond geometry (Å, °)

*D*—H⋯*A*	*D*—H	H⋯*A*	*D*⋯*A*	*D*—H⋯*A*
C11—H11*A*⋯O2	0.93	2.28	2.940 (2)	128
C14—H14*A*⋯O3^i^	0.93	2.42	3.352 (2)	175

Symmetry code: (i) 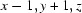.

**Fig. 1.**
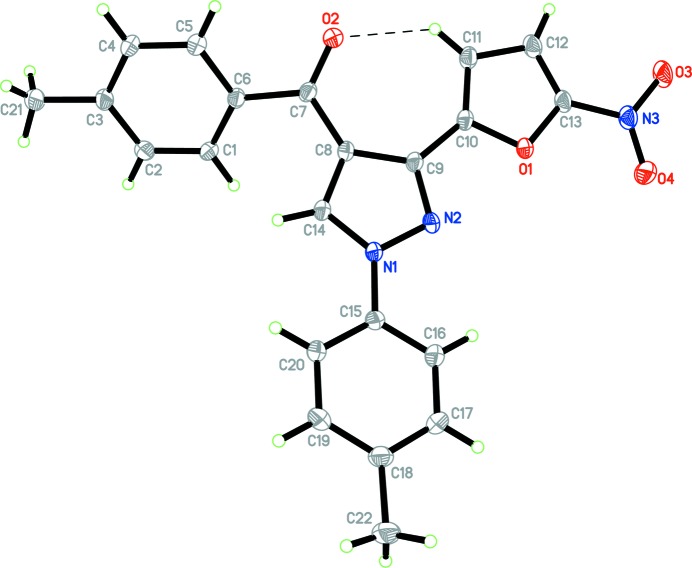
The molecular structure of (I), showing 50% probability displacement ellipsoids for non-H atoms and the atom-numbering scheme. An intramolecular C–H···O contact is shown as dashed line.

**Fig. 2.**
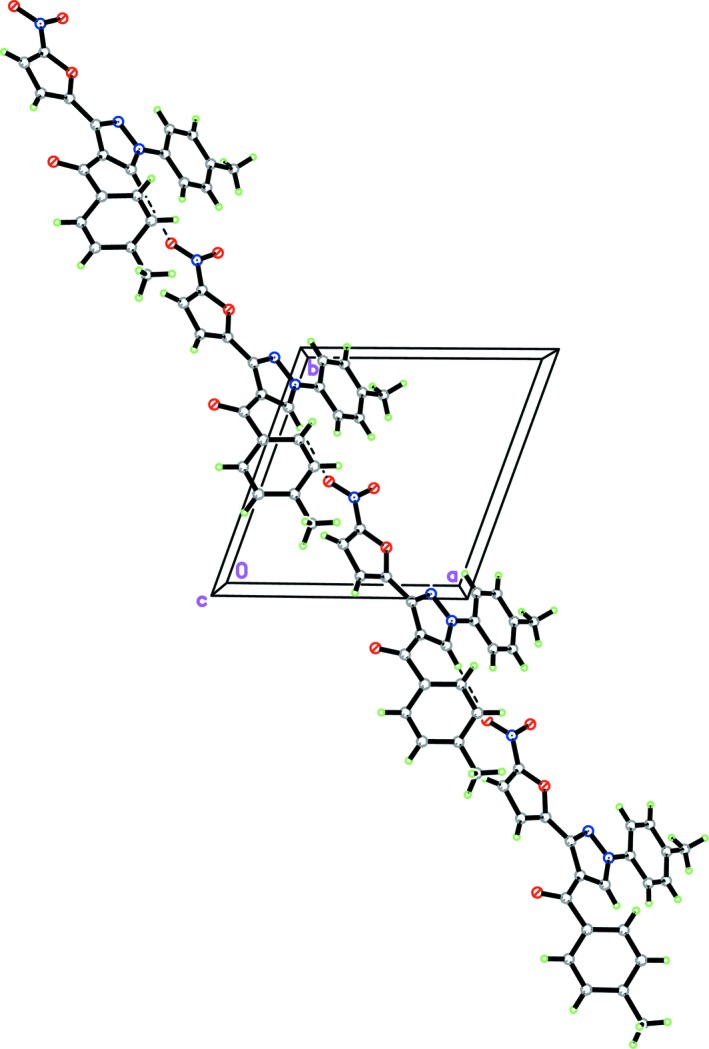
A view of the crystal structure of (I), down the c axis, showing 1-D chains along the [110] direction. Intermolecular C–H···O contacts are shown as dashed lines.

## supplementary crystallographic information

### Comment

Pyrazole derivatives are in general well-known nitrogen-containing heterocyclic compounds and various procedures have been developed for their syntheses (Rai & Kalluraya, 2006). The chemistry of pyrazole derivatives has been the subject of much interest due to their importance for various applications, and their widespread potential and proven biological and pharmacological activities (Rai *et al.*, 2008). Steroids containing pyrazole moiety are of interest as psychopharmacological agents. Some alkyl- and aryl-substituted pyrazoles have a sharply pronounced sedative action on the central nervous system. Further, certain alkyl pyrazoles show significant bacteriostatic, bacteriocidal and fungicidal, analgesic and anti-pyretic activities (Sridhar & Perumal, 2003). In continuation of our studies on 1,3-dipolar cyclo-addition reactions of sydnones with dipolarophiles carrying a nitrofuran or nitrothiophene moiety (Kalluraya *et al.*, 1994), we herein report the synthesis of this new pyrazole possessing 5-nitrofuran nucleus, (I).

In (I), an intramolecular C11—H11A···O2 contact (Table 1) generates a seven-membered ring, producing an *S*(7) ring motif (Fig. 1, Bernstein *et al.*, 1995). The furan (C10-C13/O1) and pyrazole (C8/C9/N2/N1/C14) rings are essentially planar, with maximum deviations of -0.004 (1) and 0.004 (2) Å, respectively, for atoms O1 and C9. These two rings are almost co-planar to one another, making a dihedral angle of 3.75 (10) °. One of the methylbenzene moieties (C1-C6/C21) is inclined to the pyrazole ring, as indicated by the dihedral angle formed between the mean plane through C1-C6/C21 and the C8/C9/N2/N1/C14 pyrazole ring of 48.41 (9) °. The bond lengths and angles observed are comparable to a closely related structure (Goh *et al.*, 2009).

In the crystal structure (Fig. 2), molecules are linked into a 1-D chain along the [110] direction by C14—H14A···O3 contacts (Table 1). The crystal structure is further stabilized by π–π interactions [*Cg*1···*Cg*1 = 3.4437 (10) Å; *Cg*1 is the centroid of the C8/C9/N2/N1/C14 pyrazole ring].

### Experimental

3-(p-methylphenyl)sydnone (0.01 mol) and 1-(p-methylphenyl)-3-(5-nitro-2-furyl)-2-propyn-1-one (0.01 mol) were dissolved in dry xylene (10 ml) and refluxed for 4 h. After completion of the reaction, the solvent was removed by distillation under reduced pressure. The crude product obtained was purified by recrystallization from ethanol and DMF mixture. The solid obtained was collected by filtration, washed with ethanol and dried. Single crystals were obtained by by slow evaporation of a DMF and ethanol (1:2) solution of (I).

### Refinement

All the hydrogen atoms were placed in their calculated positions, with C—H = 0.93 – 0.96 Å, and refined using a riding model, with *U*_iso_ = 1.2 or 1.5 *U*_eq_(C). A rotating group model was used for the methyl groups.

### Crystal data

**Table:**

C_22_H_17_N_3_O_4_	*Z* = 2
*M**_r_* = 387.39	*F*(000) = 404
Triclinic, *P*1	*D*_x_ = 1.406 Mg m^−^^3^
Hall symbol: -P 1	Mo *K*α radiation, λ = 0.71073 Å
*a* = 9.6398 (2) Å	Cell parameters from 9115 reflections
*b* = 9.9160 (2) Å	θ = 2.2–29.9°
*c* = 10.1815 (2) Å	µ = 0.10 mm^−^^1^
α = 88.051 (1)°	*T* = 100 K
β = 85.930 (1)°	Block, orange
γ = 70.495 (1)°	0.39 × 0.23 × 0.11 mm
*V* = 915.01 (3) Å^3^	

### Data collection

**Table:**

Bruker SMART APEXII CCD area-detector diffractometer	5261 independent reflections
Radiation source: fine-focus sealed tube	4131 reflections with *I* > 2σ(*I*)
graphite	*R*_int_ = 0.032
φ and ω scans	θ_max_ = 29.9°, θ_min_ = 2.2°
Absorption correction: multi-scan (*SADABS*; Bruker, 2005)	*h* = −13→13
*T*_min_ = 0.963, *T*_max_ = 0.989	*k* = −13→13
21316 measured reflections	*l* = −14→13

### Refinement

**Table:**

Refinement on *F*^2^	Primary atom site location: structure-invariant direct methods
Least-squares matrix: full	Secondary atom site location: difference Fourier map
*R*[*F*^2^ > 2σ(*F*^2^)] = 0.060	Hydrogen site location: inferred from neighbouring sites
*wR*(*F*^2^) = 0.144	H-atom parameters constrained
*S* = 1.08	*w* = 1/[σ^2^(*F*_o_^2^) + (0.0554*P*)^2^ + 0.6909*P*] where *P* = (*F*_o_^2^ + 2*F*_c_^2^)/3
5261 reflections	(Δ/σ)_max_ < 0.001
264 parameters	Δρ_max_ = 0.51 e Å^−^^3^
0 restraints	Δρ_min_ = −0.29 e Å^−^^3^

### Special details

**Table:**

Experimental. The crystal was placed in the cold stream of an Oxford Cyrosystems Cobra open-flow nitrogen cryostat (Cosier & Glazer, 1986) operating at 100.0 (1)K.
Geometry. All esds (except the esd in the dihedral angle between two l.s. planes) are estimated using the full covariance matrix. The cell esds are taken into account individually in the estimation of esds in distances, angles and torsion angles; correlations between esds in cell parameters are only used when they are defined by crystal symmetry. An approximate (isotropic) treatment of cell esds is used for estimating esds involving l.s. planes.
Refinement. Refinement of F^2^ against ALL reflections. The weighted R-factor wR and goodness of fit S are based on F^2^, conventional R-factors R are based on F, with F set to zero for negative F^2^. The threshold expression of F^2^ > 2sigma(F^2^) is used only for calculating R-factors(gt) etc. and is not relevant to the choice of reflections for refinement. R-factors based on F^2^ are statistically about twice as large as those based on F, and R- factors based on ALL data will be even larger.

### Fractional atomic coordinates and isotropic or equivalent isotropic displacement parameters (Å^2^)

**Table:**

	*x*	*y*	*z*	*U*_iso_*/*U*_eq_	
O1	0.37746 (12)	−0.18217 (11)	0.54625 (12)	0.0192 (3)	
O2	0.27874 (13)	0.22439 (13)	0.28781 (12)	0.0220 (3)	
O3	0.71075 (14)	−0.45933 (13)	0.52077 (13)	0.0275 (3)	
O4	0.51656 (15)	−0.43098 (14)	0.65457 (15)	0.0329 (3)	
N1	−0.00353 (15)	0.13036 (14)	0.62991 (13)	0.0165 (3)	
N2	0.12266 (15)	0.01582 (14)	0.61610 (14)	0.0173 (3)	
N3	0.58353 (16)	−0.39214 (15)	0.56224 (15)	0.0218 (3)	
C1	−0.11237 (18)	0.36641 (17)	0.26082 (16)	0.0190 (3)	
H1A	−0.1398	0.2924	0.3004	0.023*	
C2	−0.21404 (19)	0.47566 (19)	0.19334 (17)	0.0221 (4)	
H2A	−0.3088	0.4727	0.1870	0.026*	
C3	−0.17692 (19)	0.58885 (18)	0.13536 (17)	0.0227 (4)	
C4	−0.03428 (19)	0.59078 (18)	0.14428 (17)	0.0227 (4)	
H4A	−0.0079	0.6663	0.1067	0.027*	
C5	0.06906 (19)	0.48115 (17)	0.20874 (17)	0.0206 (3)	
H5A	0.1647	0.4827	0.2121	0.025*	
C6	0.03091 (18)	0.36868 (16)	0.26861 (16)	0.0171 (3)	
C7	0.15071 (18)	0.25230 (16)	0.33197 (16)	0.0169 (3)	
C8	0.11205 (17)	0.17708 (16)	0.44824 (16)	0.0162 (3)	
C9	0.19410 (17)	0.04329 (16)	0.50694 (16)	0.0160 (3)	
C10	0.33574 (18)	−0.06208 (16)	0.46636 (16)	0.0167 (3)	
C11	0.44312 (18)	−0.06981 (17)	0.36922 (16)	0.0202 (3)	
H11A	0.4408	−0.0024	0.3029	0.024*	
C12	0.55914 (19)	−0.20056 (17)	0.38871 (17)	0.0209 (3)	
H12A	0.6478	−0.2365	0.3386	0.025*	
C13	0.51276 (18)	−0.26116 (16)	0.49567 (17)	0.0192 (3)	
C14	−0.01294 (18)	0.22782 (16)	0.53221 (16)	0.0170 (3)	
H14A	−0.0897	0.3137	0.5230	0.020*	
C15	−0.10373 (17)	0.13658 (16)	0.74182 (16)	0.0164 (3)	
C16	−0.07593 (19)	0.02085 (18)	0.82857 (17)	0.0216 (3)	
H16A	0.0039	−0.0619	0.8115	0.026*	
C17	−0.1685 (2)	0.03015 (19)	0.94092 (18)	0.0246 (4)	
H17A	−0.1504	−0.0482	0.9980	0.029*	
C18	−0.28747 (19)	0.1528 (2)	0.97094 (17)	0.0227 (4)	
C19	−0.31491 (19)	0.26609 (18)	0.88030 (17)	0.0225 (4)	
H19A	−0.3948	0.3488	0.8973	0.027*	
C20	−0.22600 (18)	0.25837 (17)	0.76544 (17)	0.0194 (3)	
H20A	−0.2480	0.3338	0.7050	0.023*	
C21	−0.2876 (2)	0.7069 (2)	0.0625 (2)	0.0360 (5)	
H21A	−0.2859	0.7979	0.0900	0.054*	
H21B	−0.3844	0.7011	0.0816	0.054*	
H21C	−0.2628	0.6967	−0.0305	0.054*	
C22	−0.3809 (2)	0.1612 (2)	1.09795 (19)	0.0325 (5)	
H22A	−0.3183	0.1206	1.1685	0.049*	
H22B	−0.4475	0.1089	1.0894	0.049*	
H22C	−0.4363	0.2595	1.1167	0.049*	

### Atomic displacement parameters (Å^2^)

**Table:**

	*U*^11^	*U*^22^	*U*^33^	*U*^12^	*U*^13^	*U*^23^
O1	0.0163 (6)	0.0132 (5)	0.0240 (6)	0.0000 (4)	0.0004 (4)	0.0034 (4)
O2	0.0183 (6)	0.0197 (6)	0.0252 (6)	−0.0038 (5)	0.0025 (5)	0.0048 (5)
O3	0.0230 (6)	0.0198 (6)	0.0288 (7)	0.0067 (5)	0.0010 (5)	−0.0002 (5)
O4	0.0273 (7)	0.0218 (6)	0.0426 (8)	−0.0017 (5)	0.0048 (6)	0.0128 (6)
N1	0.0154 (6)	0.0126 (6)	0.0187 (7)	−0.0015 (5)	0.0008 (5)	0.0015 (5)
N2	0.0149 (6)	0.0121 (6)	0.0216 (7)	−0.0004 (5)	−0.0003 (5)	0.0007 (5)
N3	0.0208 (7)	0.0152 (6)	0.0251 (8)	−0.0003 (5)	−0.0016 (6)	0.0004 (5)
C1	0.0196 (8)	0.0170 (7)	0.0191 (8)	−0.0052 (6)	0.0016 (6)	0.0035 (6)
C2	0.0166 (8)	0.0251 (8)	0.0218 (8)	−0.0040 (6)	−0.0001 (6)	0.0048 (7)
C3	0.0198 (8)	0.0226 (8)	0.0198 (8)	−0.0003 (6)	0.0008 (6)	0.0064 (6)
C4	0.0242 (8)	0.0173 (7)	0.0246 (9)	−0.0053 (6)	−0.0002 (7)	0.0069 (6)
C5	0.0206 (8)	0.0179 (7)	0.0223 (8)	−0.0055 (6)	−0.0013 (6)	0.0029 (6)
C6	0.0184 (7)	0.0138 (7)	0.0169 (7)	−0.0028 (6)	−0.0004 (6)	0.0016 (6)
C7	0.0192 (8)	0.0134 (7)	0.0172 (7)	−0.0045 (6)	0.0001 (6)	0.0019 (6)
C8	0.0163 (7)	0.0122 (6)	0.0186 (8)	−0.0029 (5)	−0.0015 (6)	0.0015 (5)
C9	0.0169 (7)	0.0122 (6)	0.0176 (7)	−0.0032 (5)	−0.0009 (6)	0.0011 (5)
C10	0.0177 (7)	0.0117 (6)	0.0191 (8)	−0.0028 (6)	−0.0029 (6)	0.0013 (5)
C11	0.0210 (8)	0.0165 (7)	0.0186 (8)	−0.0007 (6)	−0.0006 (6)	0.0014 (6)
C12	0.0187 (8)	0.0182 (7)	0.0205 (8)	0.0008 (6)	0.0002 (6)	−0.0023 (6)
C13	0.0168 (8)	0.0138 (7)	0.0223 (8)	0.0010 (6)	−0.0009 (6)	−0.0005 (6)
C14	0.0175 (7)	0.0126 (6)	0.0189 (8)	−0.0024 (6)	−0.0010 (6)	0.0023 (5)
C15	0.0159 (7)	0.0155 (7)	0.0174 (7)	−0.0048 (6)	−0.0003 (6)	0.0001 (6)
C16	0.0211 (8)	0.0176 (7)	0.0227 (8)	−0.0026 (6)	−0.0003 (6)	0.0037 (6)
C17	0.0242 (9)	0.0257 (8)	0.0226 (9)	−0.0077 (7)	−0.0009 (7)	0.0079 (7)
C18	0.0197 (8)	0.0304 (9)	0.0183 (8)	−0.0090 (7)	0.0004 (6)	0.0013 (7)
C19	0.0174 (8)	0.0221 (8)	0.0251 (9)	−0.0032 (6)	0.0017 (6)	−0.0015 (7)
C20	0.0182 (8)	0.0152 (7)	0.0229 (8)	−0.0036 (6)	0.0004 (6)	0.0028 (6)
C21	0.0218 (9)	0.0388 (11)	0.0377 (11)	0.0001 (8)	0.0009 (8)	0.0226 (9)
C22	0.0253 (10)	0.0465 (12)	0.0210 (9)	−0.0070 (8)	0.0033 (7)	0.0053 (8)

### Geometric parameters (Å, °)

**Table:**

O1—C13	1.3526 (18)	C9—C10	1.457 (2)
O1—C10	1.3792 (18)	C10—C11	1.365 (2)
O2—C7	1.2262 (19)	C11—C12	1.420 (2)
O3—N3	1.2354 (18)	C11—H11A	0.9300
O4—N3	1.2264 (19)	C12—C13	1.346 (2)
N1—C14	1.349 (2)	C12—H12A	0.9300
N1—N2	1.3614 (17)	C14—H14A	0.9300
N1—C15	1.430 (2)	C15—C16	1.389 (2)
N2—C9	1.334 (2)	C15—C20	1.391 (2)
N3—C13	1.423 (2)	C16—C17	1.386 (2)
C1—C2	1.394 (2)	C16—H16A	0.9300
C1—C6	1.397 (2)	C17—C18	1.390 (2)
C1—H1A	0.9300	C17—H17A	0.9300
C2—C3	1.389 (2)	C18—C19	1.396 (2)
C2—H2A	0.9300	C18—C22	1.511 (2)
C3—C4	1.391 (3)	C19—C20	1.389 (2)
C3—C21	1.509 (2)	C19—H19A	0.9300
C4—C5	1.388 (2)	C20—H20A	0.9300
C4—H4A	0.9300	C21—H21A	0.9600
C5—C6	1.395 (2)	C21—H21B	0.9600
C5—H5A	0.9300	C21—H21C	0.9600
C6—C7	1.499 (2)	C22—H22A	0.9600
C7—C8	1.470 (2)	C22—H22B	0.9600
C8—C14	1.383 (2)	C22—H22C	0.9600
C8—C9	1.434 (2)		
			
C13—O1—C10	104.73 (12)	C12—C11—H11A	126.6
C14—N1—N2	112.14 (13)	C13—C12—C11	104.92 (14)
C14—N1—C15	128.60 (13)	C13—C12—H12A	127.5
N2—N1—C15	119.22 (13)	C11—C12—H12A	127.5
C9—N2—N1	105.09 (12)	C12—C13—O1	113.27 (14)
O4—N3—O3	124.62 (14)	C12—C13—N3	130.32 (15)
O4—N3—C13	119.26 (14)	O1—C13—N3	116.41 (14)
O3—N3—C13	116.12 (14)	N1—C14—C8	107.72 (13)
C2—C1—C6	119.62 (15)	N1—C14—H14A	126.1
C2—C1—H1A	120.2	C8—C14—H14A	126.1
C6—C1—H1A	120.2	C16—C15—C20	120.22 (15)
C3—C2—C1	121.40 (16)	C16—C15—N1	119.28 (14)
C3—C2—H2A	119.3	C20—C15—N1	120.49 (14)
C1—C2—H2A	119.3	C17—C16—C15	119.23 (15)
C2—C3—C4	118.62 (15)	C17—C16—H16A	120.4
C2—C3—C21	121.14 (17)	C15—C16—H16A	120.4
C4—C3—C21	120.24 (16)	C16—C17—C18	122.08 (16)
C5—C4—C3	120.63 (16)	C16—C17—H17A	119.0
C5—C4—H4A	119.7	C18—C17—H17A	119.0
C3—C4—H4A	119.7	C17—C18—C19	117.40 (16)
C4—C5—C6	120.65 (16)	C17—C18—C22	120.35 (16)
C4—C5—H5A	119.7	C19—C18—C22	122.25 (16)
C6—C5—H5A	119.7	C20—C19—C18	121.69 (15)
C5—C6—C1	119.07 (15)	C20—C19—H19A	119.2
C5—C6—C7	117.08 (15)	C18—C19—H19A	119.2
C1—C6—C7	123.77 (14)	C19—C20—C15	119.28 (15)
O2—C7—C8	121.55 (14)	C19—C20—H20A	120.4
O2—C7—C6	119.45 (14)	C15—C20—H20A	120.4
C8—C7—C6	118.98 (14)	C3—C21—H21A	109.5
C14—C8—C9	103.84 (13)	C3—C21—H21B	109.5
C14—C8—C7	126.10 (14)	H21A—C21—H21B	109.5
C9—C8—C7	129.94 (14)	C3—C21—H21C	109.5
N2—C9—C8	111.20 (13)	H21A—C21—H21C	109.5
N2—C9—C10	117.80 (13)	H21B—C21—H21C	109.5
C8—C9—C10	131.00 (15)	C18—C22—H22A	109.5
C11—C10—O1	110.18 (13)	C18—C22—H22B	109.5
C11—C10—C9	135.41 (15)	H22A—C22—H22B	109.5
O1—C10—C9	114.36 (13)	C18—C22—H22C	109.5
C10—C11—C12	106.90 (14)	H22A—C22—H22C	109.5
C10—C11—H11A	126.6	H22B—C22—H22C	109.5
			
C14—N1—N2—C9	0.31 (18)	C8—C9—C10—O1	−177.61 (16)
C15—N1—N2—C9	−177.78 (14)	O1—C10—C11—C12	−0.40 (19)
C6—C1—C2—C3	−1.2 (3)	C9—C10—C11—C12	176.65 (19)
C1—C2—C3—C4	0.8 (3)	C10—C11—C12—C13	0.0 (2)
C1—C2—C3—C21	−179.96 (17)	C11—C12—C13—O1	0.4 (2)
C2—C3—C4—C5	0.6 (3)	C11—C12—C13—N3	−179.11 (18)
C21—C3—C4—C5	−178.65 (17)	C10—O1—C13—C12	−0.66 (19)
C3—C4—C5—C6	−1.6 (3)	C10—O1—C13—N3	178.94 (14)
C4—C5—C6—C1	1.1 (2)	O4—N3—C13—C12	−175.80 (19)
C4—C5—C6—C7	177.95 (15)	O3—N3—C13—C12	4.0 (3)
C2—C1—C6—C5	0.2 (2)	O4—N3—C13—O1	4.7 (2)
C2—C1—C6—C7	−176.34 (15)	O3—N3—C13—O1	−175.53 (15)
C5—C6—C7—O2	−29.8 (2)	N2—N1—C14—C8	0.17 (19)
C1—C6—C7—O2	146.86 (17)	C15—N1—C14—C8	178.05 (15)
C5—C6—C7—C8	148.48 (16)	C9—C8—C14—N1	−0.54 (18)
C1—C6—C7—C8	−34.9 (2)	C7—C8—C14—N1	−176.89 (15)
O2—C7—C8—C14	156.53 (17)	C14—N1—C15—C16	177.34 (17)
C6—C7—C8—C14	−21.7 (3)	N2—N1—C15—C16	−4.9 (2)
O2—C7—C8—C9	−18.8 (3)	C14—N1—C15—C20	−4.1 (3)
C6—C7—C8—C9	162.94 (16)	N2—N1—C15—C20	173.63 (15)
N1—N2—C9—C8	−0.67 (18)	C20—C15—C16—C17	−2.2 (3)
N1—N2—C9—C10	179.90 (14)	N1—C15—C16—C17	176.38 (16)
C14—C8—C9—N2	0.77 (19)	C15—C16—C17—C18	−1.0 (3)
C7—C8—C9—N2	176.92 (16)	C16—C17—C18—C19	2.6 (3)
C14—C8—C9—C10	−179.89 (17)	C16—C17—C18—C22	−176.83 (18)
C7—C8—C9—C10	−3.7 (3)	C17—C18—C19—C20	−1.0 (3)
C13—O1—C10—C11	0.64 (18)	C22—C18—C19—C20	178.38 (18)
C13—O1—C10—C9	−177.09 (14)	C18—C19—C20—C15	−2.0 (3)
N2—C9—C10—C11	−175.27 (19)	C16—C15—C20—C19	3.7 (3)
C8—C9—C10—C11	5.4 (3)	N1—C15—C20—C19	−174.87 (16)
N2—C9—C10—O1	1.7 (2)		

### Hydrogen-bond geometry (Å, °)

**Table:**

*D*—H···*A*	*D*—H	H···*A*	*D*···*A*	*D*—H···*A*
C11—H11A···O2	0.93	2.28	2.940 (2)	128
C14—H14A···O3^i^	0.93	2.42	3.352 (2)	175

Symmetry codes: (i) *x*−1, *y*+1, *z*.
